# Measurement of Pancreatic Volume by Abdominal MRI: A Validation Study

**DOI:** 10.1371/journal.pone.0055991

**Published:** 2013-02-13

**Authors:** Edward W. Szczepaniak, Konstantinos Malliaras, Michael D. Nelson, Lidia S. Szczepaniak

**Affiliations:** Cedars-Sinai Medical Center, Los Angeles, California, United States of America; Hirosaki University Graduate School of Medicine, Japan

## Abstract

**Objective:**

To develop abdominal magnetic resonance imaging (MRI) protocol to measure pancreatic volume in humans and to validate it in large animals.

**Materials and Methods:**

We performed abdominal MRI in eight mini-pigs using a clinical 3T MRI system. We used consecutive parallel abdominal slices, covering the entire pancreas to calculate pancreatic volume. Following MRI, animals were sacrificed, the pancreas was removed, and the volume of the pancreas was measured by water displacement. We used the same MRI protocol to measure pancreatic volume in 21 humans. To assess reproducibility of in vivo measurement we repeated MRI pancreas volume evaluation within 24 hours in additional five humans.

**Results:**

In mini-pigs the measurements of pancreatic volume by MRI and by water displacement were almost identical (R^2^ = 0.9867; p<0.0001). In humans the average pancreas volume was 72.7+/−4.5 ml, range from 35.0 to 105.5 ml. This result is in strong agreement with results of previous large postmortem and computed tomography (CT) studies. Repeated measurements of pancreatic volume in humans were highly reproducible. Pancreatic volume measured in vivo was negatively correlated with age, body fat mass, pancreatic TG levels, and visceral fat mass.

**Conclusions:**

These initial results are highly encouraging and our protocol for pancreatic volume estimation in vivo may prove useful in obesity research to follow in vivo changes of pancreatic volume and structure during time course of obesity and type 2 diabetes development.

## Introduction

Magnetic resonance imaging (MRI) is routinely used for the non-invasive volume evaluation of individual organs such as the heart and the liver in humans [1]–[6]. Our objective was to develop a similar abdominal MRI protocol to measure pancreatic volume. Unlike the liver and the heart human pancreas is a small soft organ interwoven within intestines, stomach, spleen, and visceral fat. For these reasons, in vivo volumetric assessment of pancreas requires careful validation. To date, pancreatic volume assessment is limited to either post-mortem autopsy or computed tomography (CT) studies requiring radioactive agents [7]. Only handful MRI studies have been published [2], [8], [9] but none were validated. These previous human studies established that pancreatic volume changes with age and with development of metabolic diseases [7], [10], [11]. We present an abdominal MRI protocol for in vivo measurement of pancreatic volume which we validated in adult Yucatan mini-pigs and then tested in adult humans.

## Methods

The animal and human studies were approved by the Institutional Animal Care and Use Committee and by the Institutional Review Board, respectively, at Cedars - Sinai Medical Center. All human subjects gave their written informed consent to participate prior to the study.

### Animal Studies

**Figure 1:**
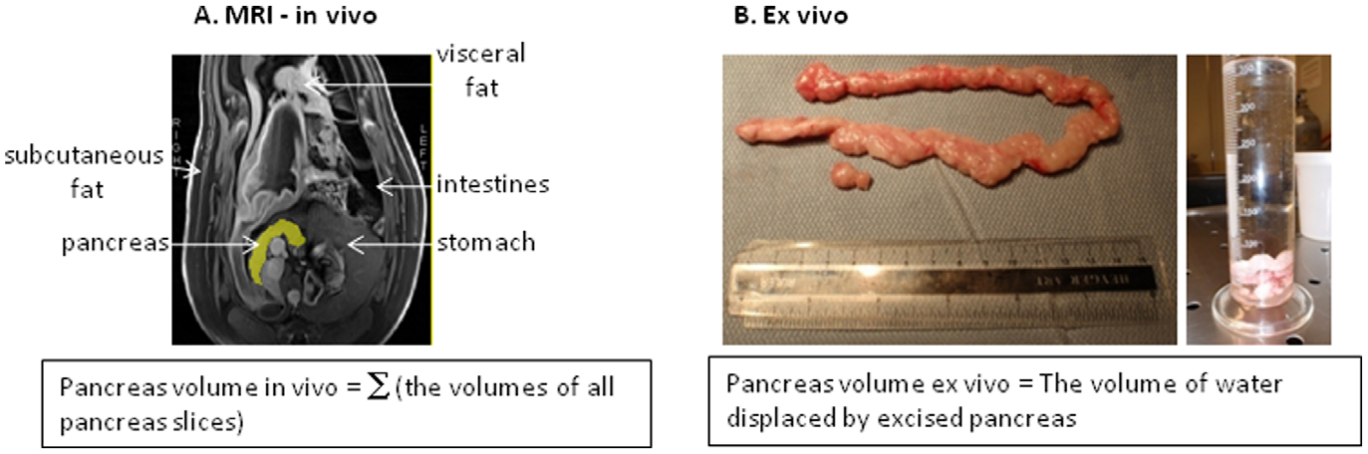
Pancreas volume measurements. A. In vivo – Representative abdominal image with highlighted pig pancreas. In average 20 abdominal slices were needed to cover entire mini-pig pancreas. B. Ex vivo - the extracted mini-pig pancreas.

We studied eight adult Yucatan mini-pigs. Prior to MR scanning animals were anesthetized with a mixture of ketamine, atropine, and acepromazine (20 mg/kg, 0.25 mg/kg, and 0.05 mg/kg respectively). Anesthesia was maintained using 2–3% isoflurane. Pig’s pancreas was imaged from “head to tail” using a 3T Siemens Verio MRI system (Siemens Medical Solutions USA, Inc.) dedicated to research in large animals and human. We used a 3D gradient echo sequence, Fast Low Angle Shot with Volume Interpolated Breath Hold (FLASH VIBE), in three perpendicular planes. We used parallel 3 mm thick contiguous slices, with a Field of View FOV = 400 mm×250 mm, inter pulse time Tr = 2.35 ms, and echo time Te = 0.96 ms. Pancreatic volume was evaluated using the DICOM image analysis software Slice-O-Matic 4.3 rev 10 from Virtual Magic, Inc., Montreal, Canada. To calculate pancreatic volume, areas of the pancreas were manually traced on oblique images, which were axial relative to the pancreas body (Figure 1). The image analysis software calculates segmental volume of each pancreatic slice as the product the slice area and thickness. The volume of the entire pancreas is then calculated as a sum of segmental volumes as indicated by the Cavalieri’s principle [4]: the volume of the pancreas in vivo = ∑ (volumes of all pancreatic slices).

After imaging, animals were sacrificed, the pancreas was removed, and the volume of the pancreas was recorded by the water displacement in a glass measuring cylinder as illustrated on Figure 1B. The in vivo and ex vivo pancreas volume measurements were than compared.

### Human Studies

We studied 21 humans of age 20–61 years. Our recruitment strategy included the following exclusion criteria: use of medication known to alter fat metabolism (i.e. steroids, pioglitazone or rosiglitazone, metformin, etc); weight loss within 6 months prior to study; any history of pancreatic or liver disorders; daily consumption of more than 2 alcoholic beverages; hypertension or other cardiovascular disease; and contraindications to magnetic resonance imaging/spectroscopy (metallic implants, claustrophobia, pregnancy, body weight above 160 kg or below 50 kg to exclude extreme obese or malnourished individuals; body circumference close to or exceeding the magnet bore size).

Subjects completed three research visits, two at the outpatient Clinical Translational Research Center (CTRC) and one at the MRI Center. The first visit included an oral glucose tolerance test, to assess glucose control status. The second visit included a frequently-sampled intravenous glucose tolerance test to assess beta-cell function and insulin-resistance. The third visit was to assess pancreatic volume with abdominal MRI and pancreatic triglyceride content with localized ^1^H MRS. All research visits were scheduled within three-weeks. In five individuals pancreatic volume measurement was repeated within 24 hours to access measurement reproducibility.

### Abdominal Magnetic Resonance Imaging (MRI)

Measurement of pancreatic volume in human was performed using the same protocol as described in the animal section. In addition to pancreatic volume we also performed MRI to measure the amount of abdominal subcutaneous and visceral fat using a single abdominal axial image at the level between the vertebral L2 and L3 bodies [12]. Image analysis was performed by a single observer blinded to the volunteer’s characteristics, using Slice-O-Matic software.

### Magnetic Resonance Spectroscopy (^1^H MRS) Measurement of Pancreatic Triglyceride

Pancreatic triglyceride (TG) levels were quantified using methods as previously described [13]. Briefly, high-resolution, perpendicular images through the abdomen were collected to locate the pancreas with the volunteer in the supine position. All images were acquired at end-expiration to suppress abdominal motion. The volume for spectroscopic testing was selected with a special attention not to include abdominal visceral fat. A large volume of interest (2 cm^3^) was used to average local pancreatic TG distribution in-homogeneities. Spectroscopic data were collected as volunteers breathed freely with the MRS signal triggered at the end-exhalation. Spectroscopic data were processed using parameters previously described [13], [14].

### Anthropometric Measurements and Clinical Evaluation

Height and weight were measured using a stadiometer and In-Body 520 scale (Biospace Co, Ltd), respectively. The In-Body scale, in addition to body weight, provided information regarding the body fat mass measured by bio-electrical impedance. In all humans systolic and diastolic blood pressure was measured using a validated oscillometric monitor with a standardized protocol [15].

### Oral Glucose Tolerance Test – OGTT

Baseline venous blood sampling was performed for measurement of lipid profile, HbA1c, and liver enzymes. A standard 75 g oral glucose tolerance test was administered to evaluate glycemic status according to American Diabetes Association criteria [16] **,** and to screen for the presence of diabetes. The test was performed at 8∶30 AM after an overnight fast. Blood was sampled at baseline and at 2 hours after glucose ingestion for evaluation of fasting and postprandial glucose and insulin levels.

### Frequently Sampled Intravenous Glucose Tolerance Test (FSIVGTT)

FSIVGTT was performed at 8∶30 AM after an overnight fast. Two intravenous polyethylene catheters were inserted into antecubital veins, one for infusion of glucose and regular human insulin and the other for blood sampling. A bolus of 50% glucose solution (0.3 g glucose/kg body weight) was injected at time 0 and a bolus of regular human insulin (0.03 U/kg body weight) was injected 20 minutes later. Blood samples were collected for determination of plasma glucose and insulin levels at −15, −10, −5, −1, 2, 3, 4, 5, 6, 8, 10, 14, 19, 22, 25, 30, 40, 50, 70, 100, 140, 180 minutes. Data were analyzed using the Millennium Minimal Model software (MINMOD) [17]. Glucose stimulated insulin secretion (AIR_g_ – acute insulin response to glucose) was measured during the first 10 minutes following the intravenous glucose bolus.

### Biochemical Analyses

Fasting plasma glucose, triglyceride, cholesterol and high density lipoprotein (HDL) concentrations as well as alanine transaminase (ALT) and aspartate transaminase (AST) were determined by enzymatic colorimetric assays using a Chemistry Analyzer Model ATAC 8000 (Elan Diagnostic) [18]. LDL cholesterol levels were calculated by Friedewald equation [19]. Plasma insulin was quantified by a paramagnetic particle chemiluminescent immunoassay using the Beckman Immunoassay Systems Access II (Beckman Coulter, Inc., Chaska, MN 55318).

### Statistical Analysis

Statistical analysis was performed using Statgraphics Centurion XVI software. In the animal study, where the pancreas volume was evaluated in vivo by MRI and ex vivo by water displacement of the excised organ, the Pearson correlation and its statistical significance were evaluated. In the human study, a simple regression with the variable transformation was used for search of relationships between pancreatic volume and other characteristics. The transformations with the highest R^2^ were selected in an attempt to find non-linear relationships. Some of those regression models showed statistical significance (p<0.05). The comparison of pancreatic volume for women and men was performed using t-test and Wilcoxon test.

## Results

**Figure 2:**
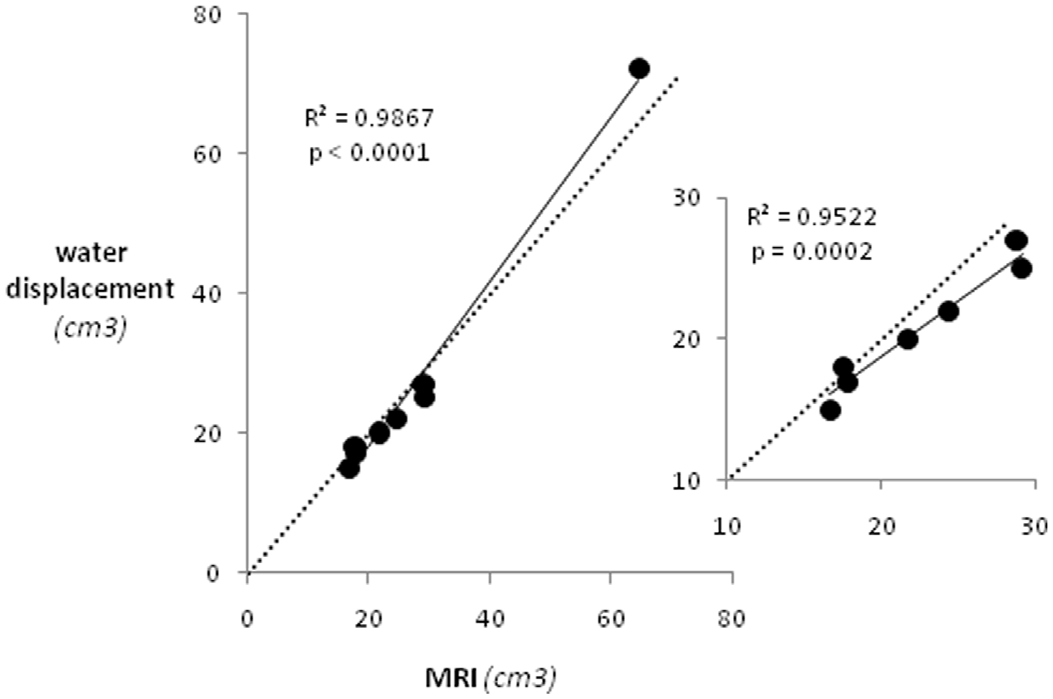
The strong correlation of pancreatic volumes measured by MRI and ex vivo. The correlation remains strong even without the point from an animal with an exceptionally large pancreatic volume. The dashed line indicates identity line.

Typically 20 MRI slices were needed to cover the entire pig’s pancreas. Figure 1A illustrates a representative MRI slice through a mini-pig abdomen with the pancreas highlighted in yellow. A representative entire pancreas removed from a mini-pig is shown in a Figure 1B. A typical pancreas was about 30 cm long and 1–2 cm wide. During pancreas removal we paid a particular attention to harvest the entire organ. The Yucatan-mini pig’s pancreas is narrow and long and it follows the lesser curvature of the stomach from the spleen to the proximal duodenum, encircling the superior mesenteric vein and extending dorsally to the left kidney [20]. Seven of the eight animals studied had pancreas of similar size, with volumes ranging from 16.7 to 29.2 ml. One animal had an exceptionally large pancreas compared to the rest despite a similar age and body weight. Pancreatic volumes measured by MRI and ex vivo correlated strongly, as illustrated in Figure 2 (R^2^ = 0.9867; p<0.0001). Importantly, exclusion of the exceptionally large pancreas did not affect the interpretation of the results, with the correlation remaining superb (R^2^ = 0.9522; p = 0.0002).

**Figure 3:**
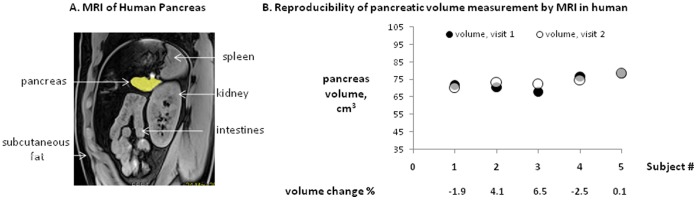
Human pancreas imaging. A. Representative, human abdominal image with pancreas highlighted in yellow. In average 47 slices were needed to cover entire human pancreas. The slice orientation is oblique relative to human body but it is axial relative to pancreas. B. High reproducibility of pancreatic volume measurement by abdominal MRI in 4 human subjects with the average pancreas size within 24 hours. The filled circles represent measurement at visit 1 and open circles represent measurement at visit 2, 24 hours later.

**Figure 4:**
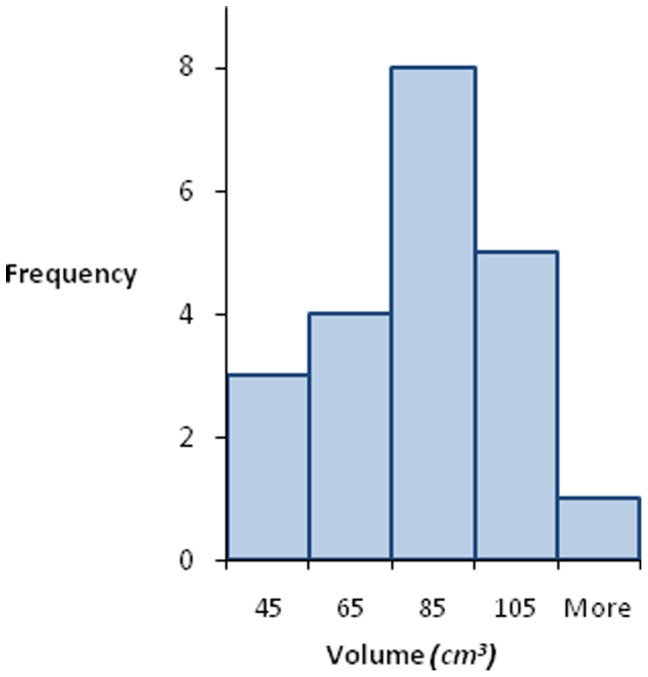
The histogram of pancreatic volume in studied population.

General characteristics, metabolic variables, abdominal fat distribution, and pancreatic triglyceride levels for human study subjects are summarized in Table1. Table 1 indicates that we studied adult, middle age humans of both genders with healthy metabolism and with normal abdominal and pancreatic fat distribution [21]. A representative abdominal image with highlighted human pancreas is shown in Figure 3A. Typically 47 oblique (axial relative to pancreas body) MRI slices were needed to cover the entire human pancreas. To assess the reproducibility of pancreatic volume measurement by MRI, we repeated measurements in five individuals within 24 hours. As illustrated in Figure 3B, reproducibility of the pancreatic volume measurement by MRI was good (percent change range (−1.9–6.5%). The distribution of pancreatic volume, in the studied population, was normal as illustrated on Figure 4. The average pancreas volume in our sample was 72.7 ml +/−4.5 ml, with a maximum volume of 105.5 ml and a minimum volume of 35.0 ml. Pancreatic volume tended to be inversely linearly correlated with age (R^2^ = 0.17; p = 0.071) and body fat mass (R^2^ = 0.29; p = 0.072). However, when transformation to non-linear relationship was allowed, pancreatic volume correlated significantly with age (R^2^ = 0.30, p = 0.011), body fat mass (R^2^ = 0.42, p = 0.023), visceral fat mass (R^2^ = 0.20, p = 0.044), and pancreatic TG levels (R^2^ = 0.23, p = 0.042). Specific non-linear relationships were:

**Table 1:** General characteristics, metabolic variables, and abdominal and pancreatic fat distribution of study subjects.

N	21	
Male, %	62	
	Mean ± Std err	Range
Age, y	40±3	20–61
BMI, *kg/m^2^*	28.0±1.2	20.3–41.3
Systolic Blood Pressure, *mmHg*	120±2	96–140
Diastolic Blood Pressure, *mmHg*	69±3	42–94
Fasting Glucose, *mg/dL*	97±3	70–124
2 Hr Glucose, *mg/dL*	129±7	55–198
Fasting Insulin, *uU/mL*	8±1	2–23
2 Hr Insulin, *uU/mL*	71±15	2–286
AIRg, mU*min/L	447±68	115–1044
Triglyceride, *mg/d*L	113±13	53–266
Cholesterol, *mg/dL*	173±8	122–244
LDL, *mg/dL*	105±6	64–151
HDL, *mg/dL*	45±3	28–96
Visceral fat, *cm^2^*	133±21	15–430
Subcutaneous fat, *cm^2^*	195±28	68–579
Pancreatic TG, *%*	5.04±1.78	0.2–25

BMI- body mass index, AIR_g_ – acute insulin response to glucose, LDL – low density lipoprotein, HDL – high density lipoprotein, SBP – systolic blood pressure, DBP – diastolic blood pressure).

(Age)^2^ = 252+103237/Pancreatic Volume;

(Body Fat Mass)^2^ = −1349+223747/Pancreas Volume;

(Visceral Fat Mass)^2^ = 24006+0.00000335/Pancreas Volume;

(Pancreatic TG)^2^ = −0.15+122/Pancreas Volume.

In our sample we did not detect gender differences in pancreatic volume nor correlation between pancreatic volume and glucose stimulated insulin secretion.

## Discussion

Our study demonstrated, that abdominal MRI allows precise, accurate, and reproducible evaluation of pancreatic volume in vivo. The correlation between mini-pig pancreatic volume measured by MRI in vivo and volumetrically post – mortem was exceptionally strong with MRI calculated volume nearly identical to true pancreatic volume measured post mortem. Additionally, pancreatic volume measurement by MRI in humans was highly reproducible. Preliminary application of our method in a sample of middle age healthy men and women yielded an average pancreatic volume of 72.7 ml +/−4.5 ml. In addition we found pancreatic volume to be reduced with increasing age, body fat mass, visceral fat mass, and pancreatic triglyceride levels. Furthermore, pancreatic volume did not differ between men and women, and was independent of acute insulin response to glucose (AIR_g_). Our results imply that the size of the pancreas can be precisely quantified by non-invasive abdominal MRI and may provide a useful tool to study the condition of the human pancreas.

Swine have been used extensively in biomedical research because they share many anatomic and physiologic characteristics of the gastro-intestinal track with humans. It has been suggested that physiologic similarities between pig and human pancreas are due to the omnivorous diet that pigs and humans consume [20]. In addition to physiological similarities the size of the Yucatan mini-pig makes it a strong comparative model to that of humans.

In the past, measurement of pancreatic volume in humans was mostly performed post mortem or using retrospective CT scanning involving radioactive agents [7]. These studies reported that the volume of the pancreas in healthy adults ranging in age from 20 to 60 years, reaches a maximum average volume of about 70 ml, to be exact 72.4 ml +/−25.8 ml [7] and 79.2+/−24.1 ml [22]. Average pancreatic volume for humans in fourth decade of life is reported as 73.9 ml +/−14.8 ml [23]. The same studies reported that pancreatic volume in healthy people declines in size with age. Our in vivo results, performed in healthy adults are in the excellent agreement with these reports even though our sample size is significantly smaller. The agreement of our results is particularly strong when comparing humans at fourth decade of life. In general, however, the literature reports are contradictory in a matter of correlations of pancreatic volume with age. As reviewed by Meier JM et al [11] some papers report significant inverse correlations between pancreatic volume and age and others report no such correlations. Indeed, in our small sample size, age only tended to be linearly related to pancreatic volume, but proved to share much stronger non-linear relationship, thus calling into question some of the previous discrepancies. Assuming good health status, it seems reasonable to assume that pancreatic volume would remain independent of age over a large portion of one’s life time (e.g. 20 to 45 years), where body mass and other physical attributes are relatively stable. We also found no gender difference in pancreatic volume, likely related to our small sample size.

The major strengths of our MRI protocol are that it is non-invasive, time efficient, and does not expose individuals to unnecessary radiation; which would be preferred for longitudinal, repeat studies and/or clinical follow-up. The fact that an experienced reader manually traced the pancreas, likely contributed to our high precision. Automatic tracing of the pancreas would be much more beneficial for large epidemiological studies, but may prove technically challenging given the non-uniform contours of the human pancreas, and may lead to increased variability. In particular, automatic tracing might miss precise outline (definition) of the traced organ, what may lead to underestimation of evaluated volume. Lastly, we acknowledge that fat infiltration in parenchymal pancreatic tissue, especially in obese individuals, is a potential confounding variable which could artificially limit this methodology. In light of these limitations we remain optimistic that the validity and reproducibility found in the present study will hold across larger populations.

In conclusion, we hope that our protocol for pancreatic volume estimation in vivo will prove useful in a variety of conditions and diseases, including obesity and type 2 diabetes, and possibly in the prevention and treatment of pancreatic cancer.
